# CD200‐CD200R1 signaling pathway regulates neuroinflammation after stroke

**DOI:** 10.1002/brb3.1882

**Published:** 2020-10-17

**Authors:** Shou‐cai Zhao, Xu Heng, Wang Ya‐ping, Luan Di, Wu Wen‐qian, Ma Ling‐song, Zhao‐hu Chu, Yang Xu

**Affiliations:** ^1^ Department of Neurology Wannan Medical College Yijishan Hospital Wuhu China; ^2^ Department of Neurology Zhu Madian Central Hospital Zhumadian China; ^3^ Department of Neurology The Second Affiliated Hospital of Wannan Medical College Wuhu China; ^4^ Key Laboratory of Non‐coding RNA Transformation Research of Anhui Higher Education Institution Wannan Medical College The First Affiliated Hospital of Wannan Medical College Wuhu China

**Keywords:** anti‐inflammatory, CD200, CD200R1, inflammation, proinflammatory, stroke

## Abstract

**Objective:**

To study how the CD200‐CD200R1 signaling pathway modulates poststroke inflammation and advances our knowledge of immune responses to ischemia insults in stroke.

**Methods:**

Focal middle cerebral artery occlusion (MCAO) was induced in mice for 90 min, and mice were sacrificed at 1, 3, and 7 days of reperfusion. CD200, CD200R1, iNOS, and Arg‐1 expression in ischemic brains was assessed by Western blotting (WB), and immunohistochemical (IHC) staining was performed to examine the expression of CD200 on neurons and CD200R1 on infiltrating lymphocytes. The severity of neurobehavioral deficits was evaluated by neurological deficit scores (NDS) and infarction volume estimated by TTC staining. To study the relationship between CD200/CD200R1 expression and the diversity of the neuroinflammatory response in stroke, CD200Fc (CD200R1 agonist) was subcutaneously injected at onset, at 1 day and 2 days after MCAO operation, and the brains were collected for detection at 3 days after MCAO/R (reperfusion).

**Results:**

CD200 expression on neurons increased at 1 day and then decreased at 3 days after MCAO/R, and the expression of CD200R1 on lymphocytes showed an opposite temporal pattern as tested by IHC. The WB results showed that CD200/CD200R1 variance exhibited a similar pattern of IHC results, and the level of iNOS peaked at 1 day and then decreased gradually, but Arg‐1 increased with time after MCAO/R in ischemic brains. After CD200Fc injection, CD200R1 expression significantly increased, and CD200Fc promoted Arg‐1 but inhibited iNOS expression. The infarct volume and NDS of the group treated with CD200Fc were significantly smaller than those of the IgG2a‐treated group.

**Conclusions:**

The CD200‐CD200R1 signaling pathway regulates neuroinflammation after stroke. Stimulation of CD200R1 by CD200Fc promotes the anti‐inflammatory response and alleviates ischemic injury.

## Graphical abstract

The CD200‐CD200R1 signal pathway is disturbed in acute stroke, and the disequilibrium of CD200‐CD200R1 signal pathway regulates the neuron‐inflammatory pathways in diversity. High expression of CD200R1 promotes the ischemic brain to release the anti‐inflammatory cytokines and mediators to relieve the ischemic injury.

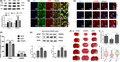

## 1 INTRODUCTION

Inflammation is widely considered an important factor in aggravated ischemic injury (Lakhan et al., 2009; Yenari et al., 2010). The initial inflammatory response evoked by the activation of microglia (MG) in the central nervous system. The rapid activation of MG is the key to initiating the inflammatory reaction in cerebral ischemia stroke (Lakhan et al., 2009; Yenari et al., 2010). Once induced by the ischemic injury, MG are activated, thus markedly enhancing the expression and secretion of cytokines and various proinflammatory factors (Gulyas et al., 2012; Perego et al., 2013).

Proinflammatory mediators, such as iNOS, MHC‐II, and CD16, decrease after ischemic injury (Cao & He, 2013); In contrast, the activated MG induced by interleukin (IL)‐4 and IL‐10, which express Arg‐1, transforming growth factor β (TGF β), IRF‐4 and so on, possess neuroprotective properties, which are considered as anti‐inflammatory cytokines (Martinez et al., 2009). In ischemic stroke, the activated MG release more anti‐inflammatory cytokines than proinflammatory cytokines in peri‐infarct regions, and as time goes on, it gradually switches to secreting more proinflammatory cytokines than anti‐inflammatory cytokines in 7 days (Hu et al., 2012; Zhao et al., 2017). However, the switch depends on the alteration of inflammatory signals with the evolution of the disease (Jimenez et al., 2008). Therefore, how to mediate inflammatory signals to elevate the anti‐inflammatory response is an important new therapeutic target that can alleviate acute stroke injury.

In the brain, the excitability of the neural network will be modified by microglia‐neuron crosstalk through regulating neuroinflammatory signals (Biber et al., 2008; Ferrini & De Koninck, 2013; de Haas et al., 2007). The interaction of CD200 with its receptor CD200R1 is critical for the downstream inhibition of proinflammatory pathways and warrants MG in a quiescent state in a physiological state (Denieffe et al., 2013; Yi et al., 2012). CD200, as a glycoprotein, is increasingly expressed on neurons (Shrivastava et al., 2012; Yi et al., 2012). A recent report has issued that CD200R1 is expressed on infiltrating lymphocytes, not on MG, and CD200R1 KO stroke brains showed higher concentrations of TNF‐α and IL‐1β in ischemic brain tissue of KO mice compared with WT controls at day 7 after stroke. Furthermore, the inhibitory signaling of CD200R1 expressed on infiltrating lymphocytes functions as a critical regulator of the peripheral immune response after brain injury, impacting mice's survival and susceptibility to poststroke infection (Ritzel et al., 2019); in contrast, the upregulation of CD200R1 can induce neuroprotective roles in inflamed brains (Hernangomez et al., 2012). The upregulation of CD200R1 would decrease IL‐1β and IL‐6 secretion and increase the level of IL‐10 in activated MG (Hernangomez et al., 2012). The role of CD200/CD200R1 signaling in regulating inflammatory has been verified in multiple sclerosis (MS) experiments, experimental autoimmune encephalomyelitis (EAE) (Chitnis et al., 2007), and in the hippocampus of aged rats (Cox et al., 2012), and it has been increasingly considered as an important modulating signaling of neuroinflammation. However, a comprehensive characterization of CD200/CD200R1 regulating the inflammatory response signals in stroke injury is still few.

In this study, we observed the expression of CD200 and CD200R1 on infiltrating lymphocytes in the brain after stroke and the diversity of the neuroinflammatory response in acute stroke. Furthermore, we used CD200Fc, a CD200R1 agonist, to investigate the effect of the CD200/CD200R1 signaling pathway on the downstream inhibitory neuroinflammatory pathway in acute stroke.

## 2 MATERIALS AND METHODS

### 2.1 Animals

Young male C57BL/6 mice were purchased from Nanjing Qinglongshan Animal Breeding Center. All experiments were performed in accordance with the National Institutes of Health Guide for the Care and Use of Laboratory and under protocols were approved by the Institutional Animal Care and Use Committee of Wannan Medical College Yijishan Hospital. The mice were group‐housed and kept on a 12:12 hr light/dark cycle, and water and rodent chow were provided freely. The utilized mice were 9–12 weeks old and 21–25 g body weight.

### 2.2 Focal cerebral middle artery occlusion/reperfusion model

The focal cerebral middle artery occlusion/reperfusion (MCAO/R) model was operated on by intraluminal occlusion of the right middle cerebral artery (MCA) for 90 min under isoflurane anesthesia as described previously (Liu et al., 2012; McCullough et al., 2005). The right MCA was occluded with 6‐0 nylon suture filaments with 0.21 mm (dimension) silicon‐coated tips. The rectal temperature was controlled at 37.0°C ± 0.5°C during surgery and the MCA occlusion (MCAO) procedure via a temperature‐regulated heating pad. Sham‐operated animals underwent the same anesthesia and surgical procedure except MCAO. In the first step, the sham, MCAO, and intervention groups were assigned randomly, and the MCAO groups were allocated to different reperfusion durations using a lottery‐drawing box. In the second step, animals in the intervention groups were randomly assigned to the sham, MCAO, MCAO + CD200Fc, and MCAO + IgG2a groups. The sham and stroke groups did not receive additional treatment after the operation; in the stroke + CD200Fc and stroke + IgG2a groups, animals were subcutaneously injected with 20 µl CD200Fc and IgG2a, respectively, at the onset of MCAO/R and 1 day and 2 days after the MCAO/R operation. At 3 days after MCAO/R, the mouse brains and blood were extracted after deep anesthesia. Investigators who were blinded to experimental group assignments performed all biochemical and histological (immunostaining and cell counting) assessments. A total of 129 mice (24 sham‐operated and 90 ischemic mice) were utilized in the study, and 15 mice were excluded from further assessments due to either death after ischemia or failure in ischemic induction.

### 2.3 Neurological behavior tests

Twenty‐four hours after MCAO/R operation, neurological deficit scores (NDS) were assessed by using a nine‐point scale (Guo et al., 2014). The scale was based on the following observations: (a) absence of neurological deficits (0 points); (b) left forelimb flexion upon suspension by the tail or failure to fully extend the right forepaw (1 point); (c) left shoulder adduction upon suspension by the tail (2 points); (d) reduced resistance to a lateral push toward the left (3 points); (e) spontaneous movement in all directions with circling to the left only if pulled by the tail (4 points); (f) circling or walking spontaneously only to the left (5 points); (g) walking only when stimulated (6 points); (h) no response to stimulation (7 points); and (i) stroke‐related death (8 points).

### 2.4 Infarct volume measurement

At the corresponding time points, mice were euthanized deeply, and the brains were removed and cut into five 2 mm slices. The slices were stained with 1.5% 2,3,4‐triphenyltetrazolium chloride (TTC) solution and incubated in a 37°C water bath for 8–10 min; the solution was changed to 4% formalin in the hood, and the slices were fixed overnight. The slice photographs were taken within 24 hr after TTC staining.

### 2.5 Immunofluorescence

Mouse brains were perfused intracardially with 1× PBS and 4% formalin; next, the brains were removed and postfixed in 4% formalin overnight. After postfixation, brains were transferred to 30% sucrose solution until equilibrated and then preserved in the −80°C freezer. After all samples were collected, the brains were cut into 30‐μm coronal sections on a freezing microtome, and the slices were then preserved in 96‐well plates filled with antifreezer solution. After washing and blocking, the brain slices were incubated with primary antibodies overnight followed by staining with secondary antibodies. Primary antibodies included rabbit anti‐CD200 (Abcam Cat# ab33734, RRID:AB_726239), rabbit anti‐CD200R1 (Thermo Fisher Cat# PA5‐47345, RRID:AB‐2605559), rat anti‐CD3 (Abcam Cat# ab16669, RRID:AB_443425), and rabbit anti‐NeuN (Abcam Cat# ab104225, RRID:AB_10711153). The secondary antibodies Cy3‐conjugated secondary antibody (Thermo Fisher Scientific Cat# A‐21203, RRID:AB_141633, Cat# A‐21207, RRID:AB_141637) and FITC‐conjugated secondary antibody (Thermo Fisher Scientific Cat# A‐11008, RRID:AB_143165, Cat# A‐11017, RRID:AB_2534084) were either goat anti‐rabbit (1:100) or goat anti‐rat (1:100) depending on the primary antibodies. Quantification of fluorescence‐positive cells was performed with ImageJ (GraphPad Software Inc, Version 6.01). Three randomly selected microscopic fields in the cortex and striatum of each section were included, and 3 consecutive sections were analyzed for each brain. Data are expressed as the mean number of cells per square millimeter.

### 2.6 Western blots

At the endpoints, mouse brain samples were obtained by rapid removal of the brain from the skull and resection of the cerebellum, followed by immediate dissection into right (ipsilateral) and left (contralateral) hemispheres. The ipsilateral hemisphere was homogenized in 1 ml RIPA buffer containing 1 mM PMSF. The extracts were immediately centrifuged at 11,300 *g* and 4°C for 15 min, and the supernatant was collected for measurement of protein concentration. The protein was subjected to Western blotting. Briefly, samples containing 30 μg protein per well were loaded onto 8%–12% SDS‐PAGE gels and transferred onto PVDF membranes (Millipore). The membranes were incubated in blocking buffer (TBST containing 5% skim milk powder) for 1 hr at room temperature and immersed in primary antibodies overnight at 4°C. The membranes were washed and subsequently incubated with the secondary antibodies for 1 hr at room temperature. Finally, an Odyssey infrared imaging system (Odyssey, LI‐COR 2800) was employed to detect the blot signals that were then quantified with Odyssey software (LI‐COR). The primary antibodies used were as follows: rabbit monoclonal antibody CD200 (Abcam Cat# ab33734, RRID:AB_726239) and rabbit polyclonal antibody CD200R1 (Thermo Fisher Cat# PA5‐47345, RRID:AB_2605559). Mouse anti‐Actin (Abcam Cat# ab8224, RRID:AB_449644) served as a loading control.

### 2.7 Statistical analysis

All data were characterized as the mean ± standard error of the mean (*SEM*), and the comparison of the means between the experimental groups was analyzed by two‐way ANOVA (with Tukey's post hoc correction for multiple comparisons where appropriate). Neurological deficit scores were analyzed with the Mann–Whitney *U* test. *p* < .05 was considered statistically significant. All in vivo and imaging studies were performed in a blinded manner.

## 3 RESULTS

1. Time course change of stroke outcomes after MCAO

**FIGURE 1:**
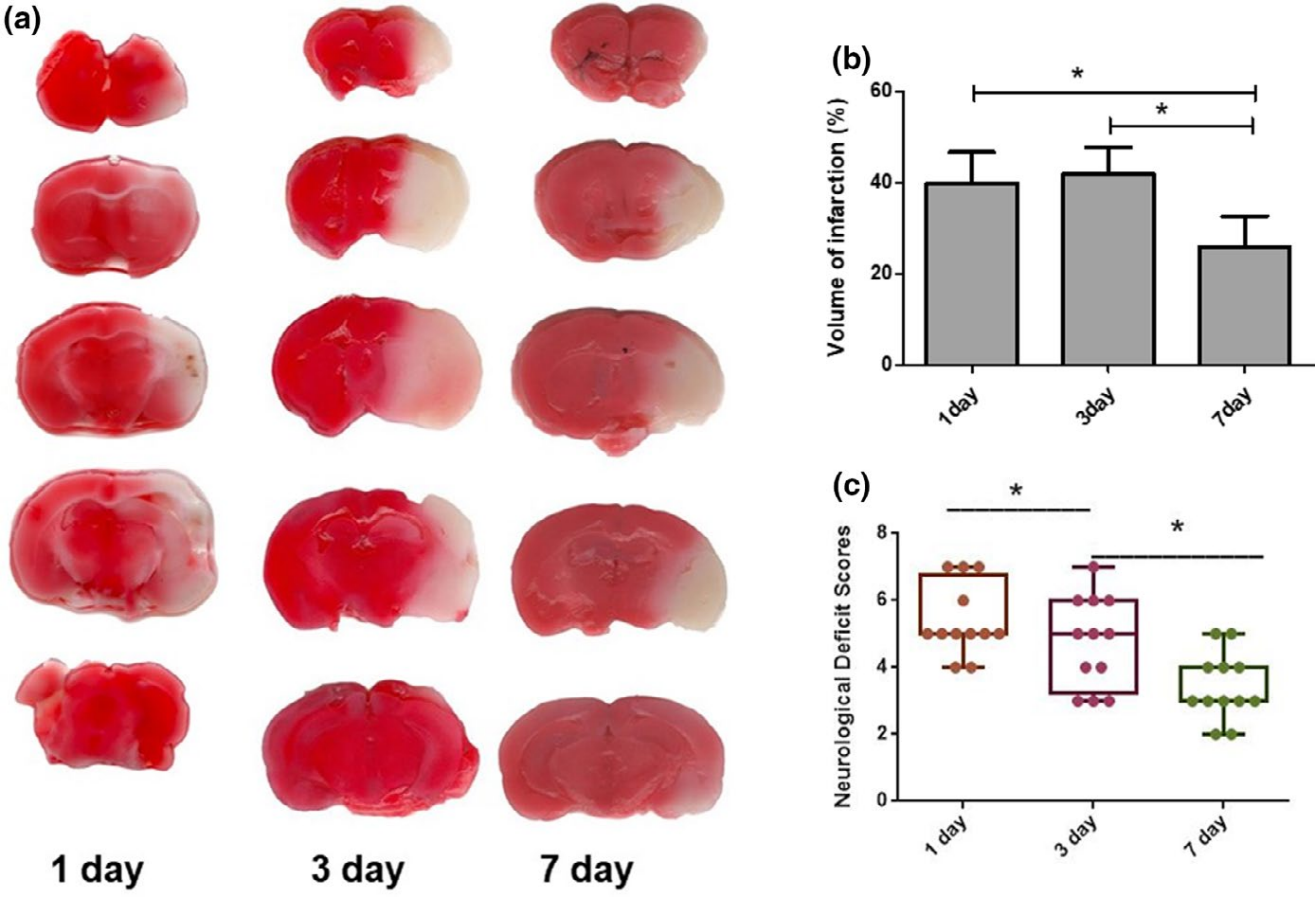
(a) 2,3,5‐Triphenyl‐2H‐tetrazolium chloride (TTC)‐stained brain of mice in different time points (*n* = 5). (b) The brain infarction volume after MCAO/R (*n* = 10). (c) The neurological deficit scores after MCAO/R (*n* = 18). The data are presented as the mean ± *SD*; *n* = 10 *p < .05

To compare outcomes after stroke at different time points, infarct volumes, and neurological behavior were examined among the 1 day, 3 days, and 7 days time points of MCAO/R in mice. The infarct size was equivalent at 1 day and 3 days of stroke mice, and a significant reduction in the infarct size was seen at day 7 in cohorts. Behavioral deficits were examined by NDS, and the results showed that the neurological deficit scores at the 1 day and 3 days time points were higher than those at the 7 days time point, while no difference existed between the 1 day and 3 days time points (Figure 1a‐c).

- CD200 expression increased at an early stage and then decreased; in contrast, CD200R1 expression increased over time after stroke

**FIGURE 2:**
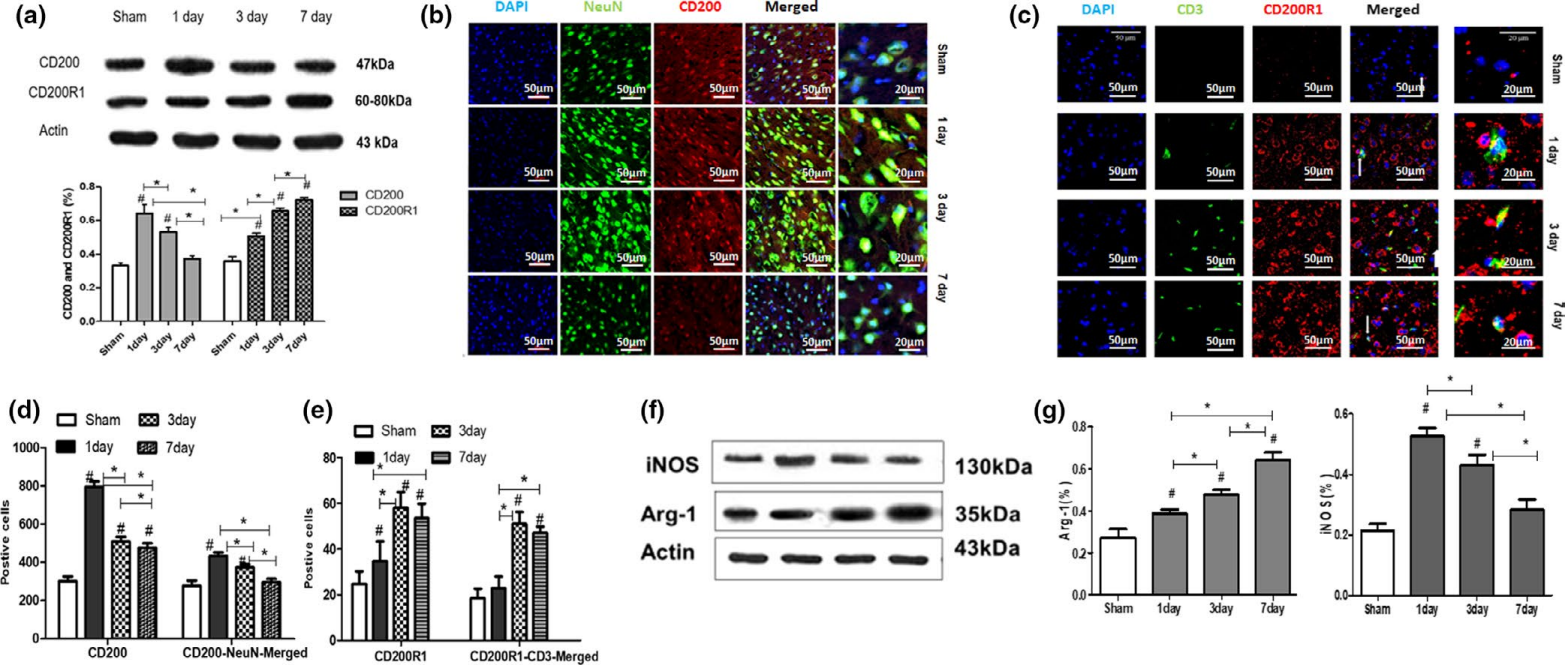
(a) CD200 and CD200R1 expression after MCAO/R by Western blot. The data are presented as the mean ± *SD*; *n* = 8 *p < .05 compared with each two subgroups, #*p* < .05 versus the Sham group. (b & d) Representative CD200 and NeuN immunofluorescence after MCAO/R. The data are presented as the mean ± *SD*; *p < .05 compared with each two subgroups, *n* = 5 #*p* < .05 versus the Sham group. (c & e) Representative CD200R1 and CD3 after MCAO/R. The data are presented as the mean ± *SD*; *n* = 5 *p < .05 compared with each two subgroups, #*p* < .05 versus the Sham group. (f & g) Representative Western blot images and quantification of the protein expression of iNOS and Arg‐1 by Western blot after MCAO/R. The data are presented as the mean ± *SD*; *n* = 5 *p < ..05 compared with each two subgroups, # *p* < .05 versus the Sham group

To investigate CD200 and CD200R1 expression changes in neurons and lymphocytes, respectively, after stroke. We performed immunofluorescence colabeling of CD200/NeuN on neurons and CD200R1/CD3 on lymphocytes to quantify CD200‐ and CD200R1‐positive cells. CD200 was expressed on neurons, and CD200R1 was expressed on lymphocytes in the ischemic cortex (Figure 2b,d). Sparse expression of CD200 or CD200R1 was observed in the sham group. In the MCAO/R groups, CD200/NeuN expression increased at 1 day and then decreased depending on the time after ischemia/reperfusion occurred. While CD200R1/CD3 expression showed opposite temporal changes, the number of positive cells increased with time over 7 days after stroke, although there was no significant difference at 3 and 7 days. The same pattern could be seen in CD200 or CD200R1 sole staining (Figure 2c,e). A temporal change in protein expression assayed by Western blots, as either CD200 or CD200R1, was similar to the immunofluorescence results in stroke groups (Figure 2a).

- Neuron‐inflammatory response exhibited the difference after stroke

Inflammation plays vital roles in secondary neuronal damage and shapes the recovery of ischemic injuries. We examined the markers of proinflammatory and anti‐inflammatory cytokine concentrations in ischemic brains with Western blots. The marker of proinflammation was identified by increased expression of iNOS, and the cytokine Arg‐1 was identified as an anti‐inflammatory marker. In the stroke group, the expression of iNOS was elevated at 1 day after MCAO/R, and dropped slowly, on the contrary, Arg‐1 expressed depended over time in 7 days after stroke (Figure 2f,g).

- CD200Fc can act as an anti‐inflammatory marker to protect neuron survival

**FIGURE 3:**
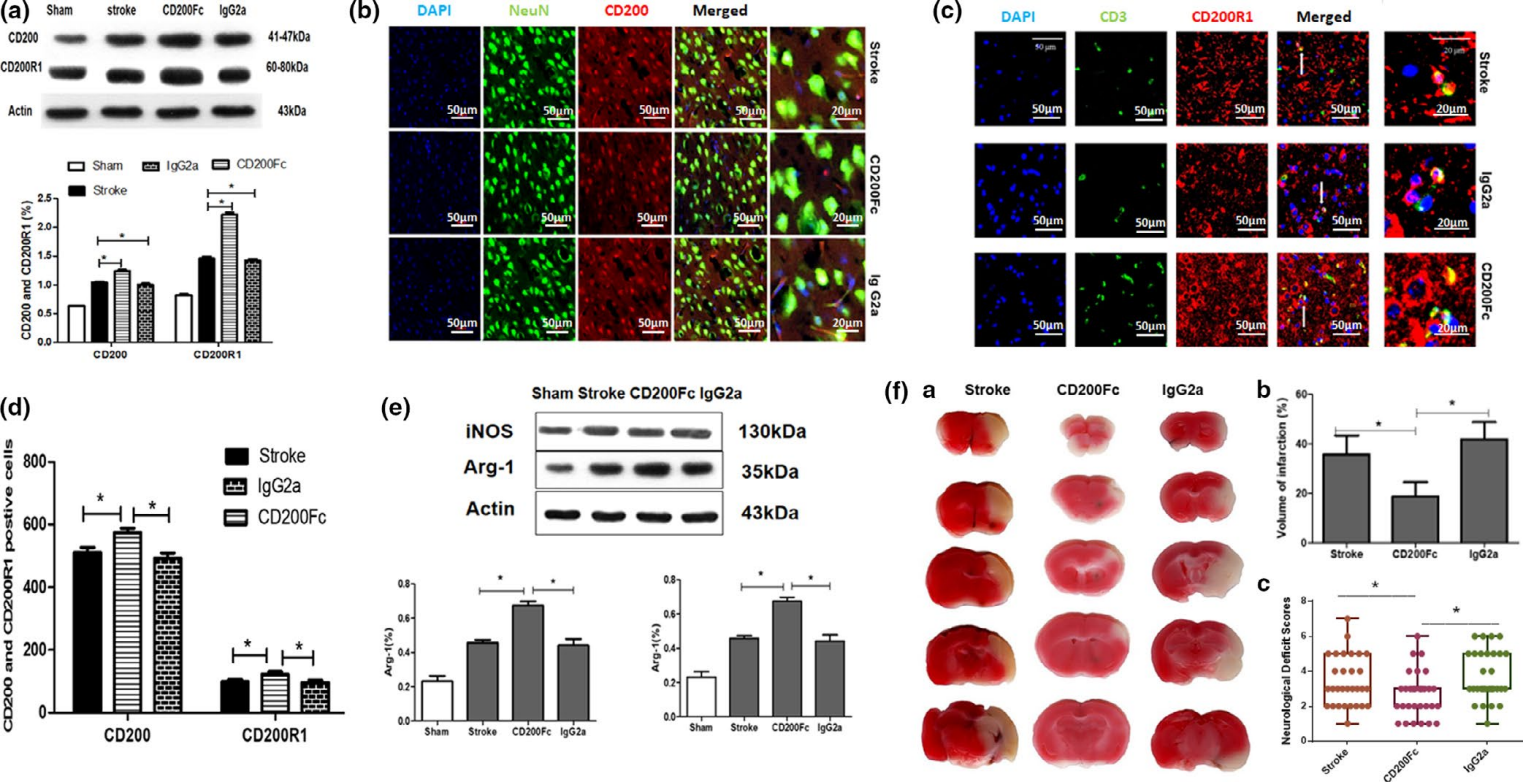
(a) CD200R and CD200R1 by Western blot at 3 day after MCAO/R; compared with each two subgroups in CD200R group and CD200R1 group. The data are presented as the mean ± *SD*; *n* = 5 *p < .05 compared with each two subgroups. (b) Representative CD200 and NeuN immunofluorescence at 3 day after MCAO/R. (c) Representative CD3 and CD200R1 immunofluorescence at 3 day after MCAO/R. (d) CD200 and CD200R1 positive cells at 3 day after MCAO/R by immunofluorescence. The data are presented as the mean ± *SD*; *n* = 5 *p < .05. (e) Representative Western blot images and quantification of the protein expression of iNOS group and Arg‐1 group at 3 day after MCAO/R by Western blot. *n* = 5 *p < .05 compared with each two subgroups. (f) The scores of neurological deficit and volume of infarction in different time points among Stroke, CD200Fc, and IgG2a groups at 3 days after MCAO/R. (a) 2,3,5‐Triphenyl‐2H‐tetrazolium chloride (TTC)‐stained brains of mice. *p < .05 compared with each two subgroups. (b) The difference of infarction volume after MCAO/R. (c) The neurological deficit scores at 24 hr after MCAO/R. *n* = 5 *p < .05 compared with each two subgroups

To investigate whether the disequilibrium of the CD200‐CD200R1 signaling pathway regulates neuron inflammation in acute stroke, mice were treated with CD200Fc, a CD200R1 agonist and injected into the peritoneal cavity after the MCAO/R model was established, and the proteins were examined at 3 days. After CD200Fc was injected, CD200R1 expression was elevated significantly, and although the expression of CD200 increased, there was no difference between CD200Fc injection and stroke in the ischemic brain after stroke (Figure 3a‐d). Neuron‐inflammation was examined by Western blots for Arg‐1 (anti‐inflammatory marker) and iNOS (proinflammatory marker). CD200Fc drove anti‐inflammatory cytokine Arg‐1 expression and inhibited the expression of the proinflammatory cytokine iNOS, whereas the injection of IgG2a did not have the same effect on neuron inflammation (Figure 3e). Furthermore, the volume of infarction in the CD200Fc group was less than that in the stroke or IgG2a group, and neurological defect scores were also similar to the change in the volume of infarction (Figure 3f).

## 4 DISCUSSION

In the pathological setting of ischemic stroke, stroke‐induced inflammatory signaling can become overactive or dysregulated, resulting in exacerbated neuronal death and loss of function (Benakis et al., 2014). The crosstalk of CD200/CD200R1 inhibitory immune ligand‐receptor could prevent the neuroinflammatory response of the brain tissue altered severely in cerebral injuries. In this study, the results showed that the dynamics of CD200 and CD200R1 expression present mismatches 7 days after stroke. The disequilibrium of the CD200‐CD200R1 signaling pathway regulated neuroinflammation variously in the ischemic brain in the acute stage after stroke. The level of anti‐inflammatory factors increased and the proinflammatory factors decreased with increasing CD200R1 expression on lymphocytes in the stroke brain. The mismatch of CD200‐CD200R1 expression was also confirmed in a glaucoma study (Taylor et al., 2011), but to date, few studies have reported it in stroke studies.

The interaction between CD200 and CD200R1 is engaged in controlling the microglial inflammatory response in a physiological state (Jurgens & Johnson, 2012; Kierdorf & Prinz, 2013; Tian et al., 2009). Emerging data have argued that CD200R1 is mainly expressed on infiltrating monocytes and lymphocytes but is absent on MG after stroke and that CD200R1 expressed in infiltrating lymphocytes is an inhibitory immune receptor in the pathological setting after stroke (Ritzel et al., 2019). CD200R1 interacting with the CD200 ligand expressed on neurons can lead to downstream inhibition of proinflammatory pathways (Hoek et al., 2000; Jenmalm et al., 2006). In this study, the expression of CD200 and CD200R1 was altered at early phases in the ischemic penumbra of stroke mouse brains. First, CD200 was expressed in ischemic neurons, and the expression of CD200R1 was in lymphocytes located in the ischemic brain after stroke. CD200 was highly expressed in the early stage and then decreased 7 days after stroke onset, whereas the expression of CD200R1 increased over time. Therefore, in acute stroke, the balance of crosstalk of the CD200‐CD200R1 signaling pathway was disturbed. Second, the stage of disequilibrium of the CD200‐CD200R1 signaling pathway modulated the inflammatory response, and the imbalance of higher expression of CD200R1 on lymphocytes and weak expression of CD200 in neurons elevated anti‐inflammatory mediator expression and descended the secretion of proinflammatory factors in the ischemic brain. In contrast, it provoked the expression of proinflammatory factors and suppressed anti‐inflammatory cytokine expression. This was confirmed by many central nervous system studies; in EAE, CD200 and CD200R1 expression was altered at presymptomatic and symptomatic phases in the central nervous system of mice that developed EAE (Valente et al., 2017). In Parkinson's disease (PD) mice, CD200 remarkably suppressed proinflammatory factors, such as IFN‐γ, TNF‐α, and IL‐1β release, and inhibited microglial activation and the release of inflammatory factors to protect dopaminergic (DA) neurons against damage and alleviate PD signs (Lyons et al., 2017; Ren et al., 2016). In aged rats, CD200Fc decreased MHC‐II and CD40 expression in the hippocampus of the brain (Cox et al., 2012). However, there are few data available on how the CD200/CD200R1 signaling pathway regulates neuronal inflammation after stroke.

Stimulation of CD200R1 by CD200 activates a signal transduction pathway that inhibits proinflammatory gene expression (Zhang et al., 2004). Previous studies in EVE have shown that the manipulation of CD200 expression or function modified the development of EAE, and its clinical signs were more severe in CD200^‐^/^‐^ than in wild‐type mice (Hoek et al., 2000); in contrast, overexpression of CD200 can attenuate the clinical signs of EAE (Chitnis et al., 2007; Meuth et al., 2008). In vitro, CD200R1 agonist treatment could inhibit the secretion of proinflammatory cytokines in activated glial cells (Hernangomez et al., 2012; Liu et al., 2010; Lyons et al., 2012). Therefore, in this study, we used the CD200R1 agonist CD200Fc to investigate changes in inflammatory cytokines after stroke. CD200Fc increased CD200R1 expression in lymphocytes, and CD200 expression in neurons also increased; at the same time, the anti‐inflammatory cytokine Arg‐1 was increasingly expressed, and the expression of iNOS, a marker of proinflammatory cytokines, was inhibited in the ischemic penumbra of the ischemic brain 7 days after stroke. These results suggest that the disequilibrium of the CD200‐CD200R1 signaling pathway regulates the pro‐/anti‐inflammatory pathways in the ischemic brain in the acute stage after stroke. However, the mechanism is unclear, and some studies have argued that CD200R1 agonists depressed the inflammatory response and inhibited disease development. The increase in CD200R1 expression compensates for the loss of function in the CD200‐CD200R1 system that follows decreased CD200 expression in EAE (Liu et al., 2010; Valente et al., 2017). One recent study argued that CD200R1 expressed on lymphocytes uniquely impacted monocyte responses, which migrated into the stroke brain from peripheral blood, to regulate the resolution of neuroinflammation in the stroke brain (Ritzel et al., 2019). Therefore, CD200R1 agonists are promising molecules that regulate neuroinflammation development in neurological disease, and CD200R1 might be a potential therapeutic target for the regulation of neuroinflammation in the acute stage of stroke.

There were several limitations to this work that we should keep in mind when the results were interpreted. We only used CD200Fc to enhance CD200R1 expression after stroke, and CD200 or CD200R1 knockout mice were not used to mechanistically study the role of the CD200‐CD200R1 signaling pathway in neuroinflammation induced by ischemic stroke.

In conclusion, the CD200‐CD200R1 signaling pathway is disturbed in acute stroke, and the disequilibrium of the CD200‐CD200R1 signaling pathway regulates the neuron‐inflammatory pathways in diversity. High expression of CD200R1 promotes the ischemic brain to release anti‐inflammatory cytokines and mediators to relieve ischemic injury.

## CONFLICT OF INTEREST

The authors declare that there is no conflict of interest.

## AUTHOR CONTRIBUTION

Zhao Shou‐cai designed the experiments; Xu Heng, Wang Ya‐ping, Luan Di, Wu Wen‐qian performed MCAO and drafted the manuscript; Chu Zhao‐hu and Ma Ling‐song performed MCAO, IHC, and Western blotting and analyzed the data; Xu Yang revised the manuscript.

### Peer Review

The peer review history for this article is available at https://publons.com/publon/10.1002/brb3.1882.

## Data Availability

The data that support the findings of this study are available from the corresponding author upon reasonable request.
